# Allogeneic Stem Cell Transplantation in Mantle Cell Lymphoma; Insights into Its Potential Role in the Era of New Immunotherapeutic and Targeted Therapies: The GETH/GELTAMO Experience

**DOI:** 10.3390/cancers14112673

**Published:** 2022-05-27

**Authors:** Antonio Gutierrez, Leyre Bento, Silvana Novelli, Alejandro Martin, Gonzalo Gutierrez, Maria Queralt Salas, Mariana Bastos-Oreiro, Ariadna Perez, Rafael Hernani, Maria Cruz Viguria, Oriana Lopez-Godino, Juan Montoro, Jose Luis Piñana, Christelle Ferra, Rocio Parody, Carmen Martin, Ignacio Español, Lucrecia Yañez, Guillermo Rodriguez, Joud Zanabili, Pilar Herrera, Maria Rosario Varela, Antonia Sampol, Carlos Solano, Dolores Caballero, on behalf of the Grupo Español de Trasplante de Progenitores Hematopoyéticos (GETH) and Grupo Español de Linfoma y Trasplante Autólogo (GELTAMO)

**Affiliations:** 1 Son Espases University HospitaI, IdISBa, 07120 Palma, Spain; leyre.bento@ssib.es (L.B.); antonia.sampolm@ssib.es (A.S.); 2 Hospital Sant Creu i Sant Pau, Service of Hematology, 08025 Barcelona, Spain; snovelli@santpau.cat; 3 Hospital Universitario Salamanca, IBSAL, CIBERONC, 37007 Salamanca, Spain; amartingar@usal.es (A.M.); cabarri@usal.es (D.C.); 4 Hospital Clinic, ICMHO, 08036 Barcelona, Spain; gonguti@clinic.cat (G.G.); mqsalas@clinic.cat (M.Q.S.); 5 Hospital General Universitario Gregorio Marañón, 28007 Madrid, Spain; marianabeatriz.bastos@salud.madrid.org; 6 Hospital Clínico Valencia, 46010 Valencia, Spain; ariadna_pema@hotmail.com (A.P.); rafa_hm@hotmail.com (R.H.); carlos.solano@uv.es (C.S.); 7 Complejo Hospitalario de Navarra, 31008 Pamplona, Spain; mc.viguria.alegria@navarra.es; 8 Hospital Morales Meseguer, 30008 Murcia, Spain; orilopezgodino@gmail.com; 9 Hospital La Fe, Facultad de Medicina, Universidad Catolica de Valencia, 46026 Valencia, Spain; juanmontorogomez@gmail.com (J.M.); jlpinana@gmail.com (J.L.P.); 10 ICO-IJC-Hospital Germans Trias i Pujol, 08916 Badalona, Spain; cferra@iconcologia.net; 11 Institut Català d’Oncologia (ICO), 08908 L’Hospitalet de Llobregat, Spain; rparody@iconcologia.net; 12 Hospital Reina Sofía, 14004 Córdoba, Spain; carmen.martin.calvo.sspa@juntadeandalucia.es; 13 Hospital Universitario Virgen de Arrixaca, 30120 Murcia, Spain; i.espanol@gmail.com; 14 Hospital Marques de Valdecilla, IDIVAL, 39008 Santander, Spain; lucrecia.yanez@scsalud.es; 15 Hospital Virgen del Rocío, 41013 Sevilla, Spain; grgarcia@gmail.com; 16 Hospital Asturias, 33011 Oviedo, Spain; joudzas88@gmail.com; 17 Hospital Ramón y Cajal, 28034 Madrid, Spain; pherrera.hrc@gmail.com; 18 Complejo Hospital Juan Canalejo, 15006 Coruña, Spain; ma.rosario.varela.gomez@sergas.es

**Keywords:** mantle cell lymphoma, allogeneic stem-cell transplantation, non-relapse mortality, acute graft-versus-host disease, graft-versus-lymphoma effect, target therapy, CAR-T cell therapy

## Abstract

**Simple Summary:**

We present the long-term results of patients receiving allogeneic stem cell transplantation (allo-SCT) for relapsed/refractory mantle cell lymphoma (R/R MCL) in the last 25 years in Spain. We conclude that allo-SCT may be a curative option in R/R MCL with a low cumulative incidence (CI) of relapse, although non-relapse mortality (NRM) is still high, which is mainly secondary to acute graft-versus-host disease (aGVHD). Results are better for fit patients, using HLA-identical (related or unrelated) or haploidentical related donors and without previous ASCT. However, the arrival of new highly effective and low toxic immunotherapeutic or targeted therapies inevitably will relegate allo-SCT to those fit patients who fail these therapies, being administered far away from the optimal timing.

**Abstract:**

Allo-SCT is a curative option for selected patients with relapsed/refractory (R/R) MCL, but with significant NRM. We present the long-term results of patients receiving allo-SCT in Spain from March 1995 to February 2020. The primary endpoints were EFS, OS, and cumulative incidence (CI) of NRM, relapse, and GVHD. We included 135 patients, most (85%) receiving RIC. After a median follow-up of 68 months, 5-year EFS and OS were 47 and 50%, respectively. Overall and CR rates were 86 and 80%. The CI of relapse at 1 and 3 years were 7 and 12%. NRM at day 100 and 1 year were 17 and 32%. Previous ASCT and Grade 3–4 aGVHD were associated with a higher NRM. Grade 3–4 aGVHD, donor type (mismatch non-related), and the time-period 2006–2020 were independently related to worse EFS. Patients from 1995–2005 were younger, most from HLA-identical sibling donors, and were pretreated less. Our data confirmed that allo-SCT may be a curative option in R/R MCL with low a CI of relapse, although NRM is still high, being mainly secondary to aGVHD. The arrival of new, highly effective and low toxic immunotherapeutic or targeted therapies inevitably will relegate allo-SCT to those fit patients who fail these therapies, far away from the optimal timing of treatment.

## 1. Introduction

Mantle cell lymphoma (MCL) is an uncommon B-cell lymphoma that generally has a poor prognosis, with high rates of chemorefractoriness and an advanced median age at diagnosis [1]. For transplant-eligible patients, clinical outcome improves using intensive cytarabine-based induction chemotherapy, followed by autologous stem cell transplant (ASCT) [2,3] consolidation. Recently, new approaches with anti-CD20 maintenance [4,5], new target drugs [6,7], or new ways of immunotherapy such as CARTs [8] are changing the front and salvage therapeutic lines.

Allogeneic stem cell transplantation (allo-SCT) is a potential curative option for selected patients, mediated by a well-demonstrated graft versus lymphoma (GVL) effect in this lymphoma [9]. However, allo-SCT in MCL has also been associated with significant non-relapse mortality (NRM) [10,11]. The efficacy and toxicity of allo-SCT should be well-balanced, considering that MCL typically affects an older population with potentially higher rates of comorbidities, and there is an increasing number of emerging effective and manageable alternative therapeutic approaches. For these reasons, there is a need to clarify candidates’ selection, and which is the optimal target population for allo-SCT. We should especially consider that most previously published studies that focused on investigating the role of allo-SCT in MCL are retrospective and conclusions are limited by a reduced sample size of patients. Furthermore, this is particularly important with the outstanding efficacy and manageable toxicity associated with new immunotherapies based on CAR-Ts in relapsed/refractory (R/R) MCL [8,12].

Our objective was to analyze the long-term results of MCL patients undergoing allo-SCT in Spain, trying to define better its current role in the era of new immunotherapeutic and targeted therapies, focusing on candidates’ selection.

## 2. Materials and Methods

### 2.1. Study Design and Patient Eligibility

We designed a retrospective multicenter study including all registered patients from centers’ members of the Spanish Group of Hematopoietic Transplantation (GETH)/Spanish Group of Lymphoma (GELTAMO) with relapsed or refractory (R/R) MCL consolidated with allo-SCT. For this purpose, all patients who had undergone an allo-SCT in the abovementioned centers from March 1995 to February 2020 that was reported to the EBMT registry were eligible. The primary endpoints were event-free survival (EFS) and overall survival (OS). Secondary outcomes were cumulative incidence (CI) of NRM, relapse, and graft versus host disease (GVHD). The study was performed in compliance with the Declaration of Helsinki and approved by research ethics committees and institutional review boards at each participating institution. As part of the EBMT registration, all patients signed informed consent.

### 2.2. Data Recovery and Study Definitions

The histological diagnosis was based on a local review, and patients were staged according to the Ann Arbor system. Disease status was assessed by the local team according to the Revised Response Criteria for Malignant Lymphoma [13] and/or Lugano Classification [14]. Myeloablative conditioning was defined as a regimen containing either total body irradiation (TBI) with a dose greater than 6 Gy, a total dose of oral busulfan greater than 8 mg/kg, or a total dose of intravenous busulfan greater than 6.4 mg/kg. All other regimens were defined as reduced-intensity conditioning (RIC) [15]. The diagnosis and grading of acute and chronic graft versus host disease (aGVHD and cGVHD) were performed by the transplant centers using the standard criteria [16].

### 2.3. Statistical Analysis

All outcome measures were assessed from the time of allo-SCT. OS was defined as the time to death. Event-free survival (EFS) was defined as the time to relapse, progression, or death from any cause. NRM was defined as the time to death without previous disease relapse or progression (considering relapse as a competing event). CI of relapse was defined as the time from relapse or progression (considering death without relapse as a competing event).

Qualitative or binomial variables are expressed as frequencies and percentages. Comparisons between qualitative variables were made using the Fisher Exact Test or the Chi-squared test. Comparisons between quantitative and qualitative variables were performed through non-parametric tests (U of Mann–Whitney or Kruskal–Wallis). The binary logistic regression was used to find out the risk factors associated with day 100 complete response (CR) or NRM. Time to event variables were estimated according to the Kaplan–Meier method and comparisons between variables of interest were performed by the log-rank test. Multivariate analysis with the variables that appeared to be significant in the univariate analysis was carried out according to the Cox proportional hazard regression model (stepwise forward likelihood ratio selection). Those variables not available at transplant were included as time-dependent variables. All *p* values reported were 2-sided, and statistical significance was defined at *p* < 0.05. To analyze the impact of time periods on the survival of transplanted patients, we segmented the full range of follow-up (1995–2020) of our series using MAXTAT for disease progression or death. The statistical analysis was performed using SPSS software (SPSS version 28.0; IBM, Chicago, IL, USA) and RStudio (Version 1.3.959; RStudio, PBC, Boston, MA, USA).

## 3. Results

### 3.1. Patient and Allo-SCT Characteristics

A total of 135 patients with R/R MCL that fulfilled the inclusion criteria were included in the study. Table 1 summarizes the main patient and allo-SCT information. Briefly, the median age of the study cohort at the time of the allo-SCT was 56 years (32–70), with 27% of patients being older than 60 years. A proportion of 66% of patients had classic, 27% blastic, and 7% indolent MCL. The median time from diagnosis to allo-SCT was 33 months (3–164), and the median number of previous lines of therapy before allo-SCT was two (one to eight), including previous autologous SCT (ASCT) in 49% of patients. Disease status before allo-SCT was as follows: complete response (CR) in 86 patients (64%), partial response (PR) in 35 (26%) patients, and SD/PD in 13 (10%).

Overall, 85% adults underwent RIC allo-SCT, and most patients received grafts from HLA-matched, related and unrelated donors (76%) followed by haploidentical donors (13%), and 9/10 mismatched unrelated donors (10%).

### 3.2. Main Outcome Data

**Figure 1:**
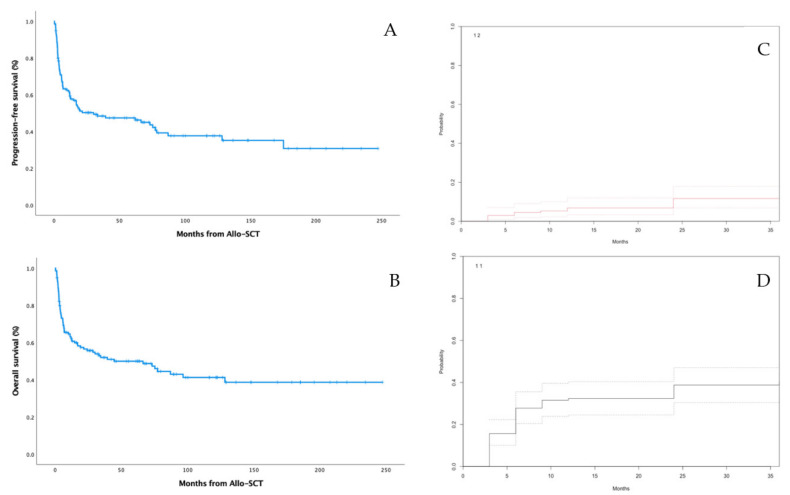
Main outcome data of the patients including event-free survival (**A**), overall survival (**B**), CI of relapse (**C**)and CI of NRM (**D**).

After a median follow-up of 68 months (2–247), median EFS and OS were 30 (95% CI: 0–72) and 45 (95% CI: 3–86) months, respectively (Figure 1A,B). Eighteen (13%) patients had a progression of lymphoma and 71 (53%) died. Overall and complete response rates (ORR and CR) at day 100 were 84 and 80%, respectively. Most patients (95%) with CR before allo-SCT maintained CR at day 100, while only 2 and 1%, respectively, had a PR or a disease progression. On the other hand, 73 and 50% of patients with previous PR or SD/PD achieved CR at day 100, with only 9 and 17%, respectively, showing progressive disease at day 100. Factors that were significantly associated with CR at day 100 were CR pre-allo-SCT (relative risk (RR) 9.6; 95% CI: 2.9–31.3; *p* < 0.001) and less than three prior lines (RR 3.1; 95% CI: 1.1–8.9; *p* = 0.036). The CIs of relapse at 1 and 3 years were 7% (95% CI: 3–12) and 12% (95% CI: 7–18), respectively (Figure 1C).

### 3.3. GVHD and NRM

Seventy-four (55%) patients developed aGVHD (Grade 1–2: 42 (31%) and Grade 3–4: 32 (24%)) at a median of 31 days post-allo-SCT. The CI of overall acute, acute Grade 2-4, and acute Grade 3-4 GVHD at day 100 were 53% (95% CI: 45–62), 48% (95% CI: 39–57), and 29% (95% CI: 62–80), respectively. Forty-eight (36%) patients developed cGVHD (13% mild, 11% moderate, 10% severe). The CIs of overall chronic and chronic moderate/severe GVHD at 3 years were 57% (CI 95%: 45–68) and 42% (CI 95%: 29–55), respectively.

The CIs of day 100 and one-year NRM were 17% (95% CI: 11–24) and 32% (95% CI: 24–40), respectively (Figure 1D). When analyzing all pretransplant factors included in Table 1, as well as aGVHD incidence, to assess their contribution to NRM at day 100, we observed that patients having a previous ASCT (RR 3; 95% CI: 1.1–8.1; *p* = 0.03) and those who developed Grade 3–4 aGVHD (RR 5.4; 95% CI: 2.1–13.7; *p* < 0.001) were significantly and independently related to a higher NRM. In contrast, the NRM was not influenced by age, ECOG PS, conditioning regimen, or donor type (Table 2).

### 3.4. Survival Analysis

One and 5-year-EFS were 61% (95% CI: 57–65) and 47% (95% CI: 39–56), respectively. On the other hand, one and 5-year-OS were 63% (95% CI: 59–67) and 50% (95% CI: 41–59), respectively. Univariate analysis showed that EFS and OS were influenced by the type of donor (mismatch), ECOG PS at allo-SCT, pre-transplant response, GVHD prophylaxis, response to allo-SCT, aGVHD, cGVHD, and time-period (Table 3). In the multivariate analysis, three variables showed an independent prognostic value for a worse EFS: grade 3–4 aGVHD, donor type (mismatch non-related), and time period 2006–2020.

The main causes of death were associated with NRM: 32 due to GVHD (45%), most of them were grade 3–4, 15 to infections (21%), 2 sinusoidal obstruction syndromes (SOS) (3%), 2 thrombotic microangiopathy (3%) and 8 other causes (11%). Progression of lymphoma was the cause of death in 12 (17%) patients. Most patients suffering NRM were in CR (69%), 8% in PR and 22% died of NRM before response evaluation.

### 3.5. Impact in Results of Allo-SCT Candidates’ Selection along Decades

**Figure 2:**
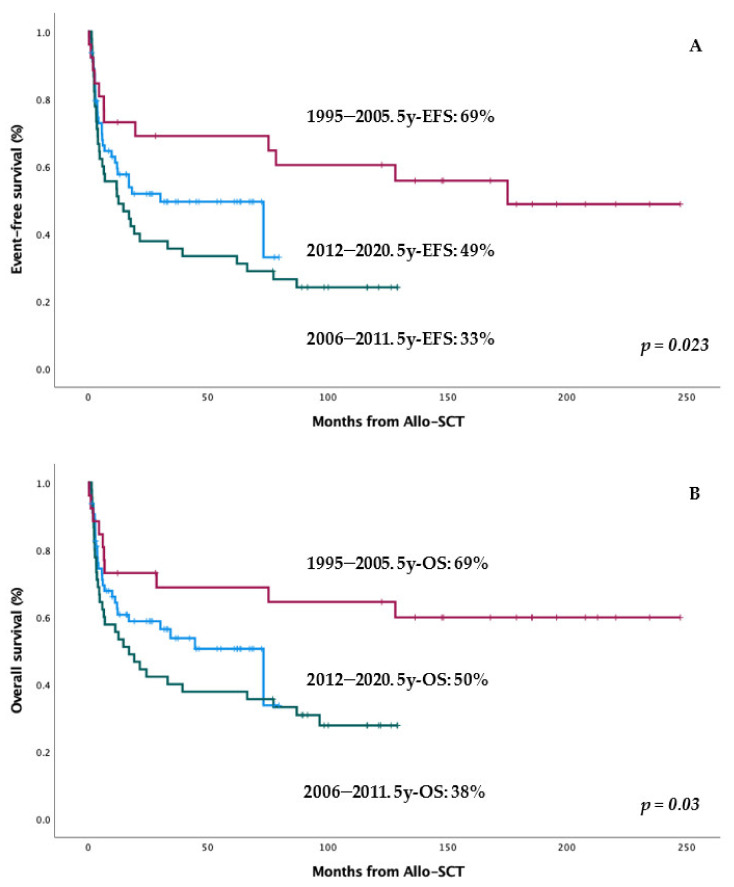
Impact of time-periods in EFS (**A**) and OS (**B**) after allo-SCT.

We focused on the impact of time periods on the survival of transplanted patients. For this purpose, we segmented the full range of follow-up (1995–2020) of our series using MAXTAT for EFS, obtaining two cutoffs at 15 and 9 years: 2005 and 2011. As shown in Table 3 and Figure 2, the best outcomes in terms of EFS were obtained from 1995 to 2005; with the worst observed from 2006 to 2011. Since 2012, the results slightly improved again but still did not achieve previous levels. However, the 5-year CIs of relapse were similar between all three time periods from 1995 to 2020: 14, 15, and 19% (*p* = 0.89).

We compared patient characteristics along time periods to discover the causes of these results. As shown in Table 4, patients from 1995 to 2005 were younger, with a much shorter interval from diagnosis to allo-SCT; most of them were from HLA-identical sibling donors and with much less previous therapy. In other words, from 1995 to 2005, patients were much more selected for and transplanted earlier than after 2005. This translated into a lower NRM and a better EFS and OS in these patients (Table 3 and Table 4).

## 4. Discussion

Our study presents real-world evidence from GETH and GELTAMO Spanish centers about the role of allo-SCT in MCL, confirming its efficacy as a potentially curative option but, at the same time, highlighting its major handicap in terms of potential toxicity and high NRM. However, the most important point is that our series provides a great insight into the importance of candidates’ selection for allo-SCT, which limits its current role in the era of new immunotherapeutic and targeted therapies in MCL.

Most previously published series about the role of allo-SCT in MCL are retrospective and generally small. Our retrospective series with 135 patients compares favorably with most of them in terms of size. It was obtained from all patients reported to the EBMT registry from Spanish centers, with a 5-year EFS and OS of 47 and 50%, respectively. This is in the range between 30–60% of the previously published studies [9,18,19].

All these works and our series provide convincing evidence of the existence of an allogeneic GVL effect, suggesting a curative potential, although this is weaker than in indolent lymphoma [9,18,19,20]. This is also illustrated by the better EFS in patients having cGVHD and the high number of responses in our patients: overall 80% CR, which was higher in patients with previous CR (95%) but also in cases with previous PR (73%) or even SD/PD (50%), demonstrating the graft-versus-MCL effect. Furthermore, the incidence of cGVHD was significantly higher in those patients with less than a CR at pretransplant, who then obtained a CR posttransplant (43%), vs those not achieving a CR (0%) (*p* = 0.027). Other works have reported that chemorefractoriness is not a major risk factor for disease control in MCL after allo-SCT [21]. In our series, the relapse rate was not a major challenge (only 7 and 12% at 1 and 3 years, respectively). This contrasts with other RIC series in which relapse was reported in up to 40% [18]. However, the high rate of NRM might reduce the number of patients at risk of relapse in our series.

Most previously published studies in allo-SCT in MCL share high rates of toxicity in terms of 10 to more than 50% of NRM, as well as high rates (30–40%) of acute or chronic GVHD. Of course, the higher rates of NRM have been reported to be associated with myeloablative conditioning regimens as well as in more pretreated patients, particularly those failing ASCT [10,11], as was seen in our patients. These high rates of NRM may be lower (below 30%) with a similar efficacy using RIC, as shown in several retrospective [18,19,21,22,23] or even prospective clinical trials [24]. New strategies of GVHD prophylaxis such as cyclophosphamide post-allo-SCT could also improve NRM results in these patients [25,26]. However, only 9% of our patients received this prophylaxis in some of our last haploidentical transplants. In our series, we had 17 and 32% CI of NRM at 100 days and 1 year, respectively, which was independently associated with Grade 3–4 aGVHD incidence and previous ASCT, but not with other transplant characteristics. This may be influenced by the fact that most of our patients received an RIC regimen (85%).

For these reasons, once one demonstrates efficacy, this should be balanced against significant rates of NRM, and is when patient selection and other therapeutic options should be considered. In our series, we observed worse outcome results in terms of EFS and OS in patients with Grade 3–4 aGVHD who were transplanted with unrelated mismatched donors (related mismatched transplants, including haploidentical ones, fared similarly to HLA-identical procedures) and, unexpectedly, in the period 2006 to 2020, this was clearly related to a change in the pattern of candidate selection towards older and more pretreated patients beyond 2006. This is the logical consequence of having much better alternatives to allo-SCT in this population of patients, with also better results.

Nowadays, frontline high-dose cytarabine-containing programs followed by ASCT have been considered the standard of care for young and fit patients [3,4]. When comparing the abovementioned time-periods, in the older cohort (1995–2005), less than half of the patients received an ASCT (19 vs. 56% beyond 2006) as the value of frontline ASCT in MCL was first reported in 2005 [2]. Maintenance with rituximab has been shown to prolong PFS and OS, both in fit or unfit patients [4,5]. New, non-cross resistant chemotherapeutic drugs have shown interesting activity in MCL such as oxaliplatin [27,28,29], bortezomib [30], or bendamustine [31,32,33]. Targeted therapies such as BTK inhibitors [6,34,35] or venetoclax [7,36] obtain impressive results with manageable toxicity, representing good salvage options that may also delay the decision of allo-SCT. Finally, outstanding results have been reported with new immunotherapies with anti-CD19 CAR-T [8,12], which led to the approval of brexucabtagene autoleucel as salvage therapy for R/R MCL patients. Points favoring CAR-T cell therapy are that it is more effective than allo-SCT in patients with active disease, with much lower rates of toxicity and NRM. On the other hand, the follow-up with CAR-T cell therapy is still short when compared with allo-SCT, so it is not known for its long-term curative potential; there is also a relevant economic impact, and limited accessibility. Considering the sequencing of both approaches, if CAR-T cell therapy fails, patients could still receive an allo-SCT but in a more pretreated status that we know would further reduce the efficacy and increase the toxicity of this procedure.

The most important updated guidelines from the American Society of Transplantation and Cellular Therapy, Center of International Blood and Marrow Transplant Research, European Society for Blood and Marrow Transplantation [37], and National Comprehensive Cancer Network (NCCN) recommend CAR-T cell therapies in MCL from the third line for patients who are intolerant to or relapse after at least one BTKi. NCCN guidelines also recommend considering allo-SCT as consolidation for high-risk, young, and fit responders to a second line. Consequently, if there is an increase in the accessibility of CAR-T cell therapy, we hypothesize that CAR-T cell therapies will probably delay allo-SCT to later lines of therapy, far away from the optimal timing evidenced in our series.

Our work has several limitations. First, it is a retrospective study that implies real world evidence but lacks homogeneity in terms of frontline or salvage therapies, supportive care strategies, and GVHD prophylaxis and management. We do not have some important diagnostic or prognostic information, such as molecular high-risk profiles or TP53 mutation status that could modify therapeutic decisions for allo-SCT candidates [38,39]. Addressing the abovementioned lack of some new strategies for GVHD prophylaxis such as cyclophosphamide post-allo-SCT, we could also mention a few patients receiving ibrutinib before and after allo-SCT, which may provide a benefit in terms of survival [40]. However, in our series, we only had 12 (9%) patients with cyclophosphamide post-allo-SCT prophylaxis and 19 (10%) having received ibrutinib.

Of note, while the older cohort (1995–2005) comprised essentially of identical siblings in contrast with the later cohorts, GVHD incidence was not significantly different and Grade 3–4 aGVHD was only 10% lower in the older cohort. This may illustrate the improvement in GVHD prophylaxis and therapy with the time. At the same time, in our study there were several patients at a high risk for poor outcomes, in which allo-SCT usually is contraindicated: ECOG PS 2–4 (5%) and pretransplant SD/PD (10%). In our series, 5 y-PFS was respectively 0 and 15%, illustrating the potential for disease control of allo-SCT even in selected cases with active disease at transplant, as well as the need of a good ECOG PS to avoid NRM.

## 5. Conclusions

Taking all this data together, allo-SCT is a feasible and effective therapy in MCL with a well-demonstrated GVL effect favored by cGVHD even in situations of active disease at allo-SCT, but still with high rates of toxicity and NRM. As concluded from our time-period analysis, allo-SCT may be a better approach for young, fit, high-risk patients consolidated early (i.e., second line) that probably is linked to lower rates of severe aGVHD and NRM. Improved outcomes may be obtained using HLA-identical (related or unrelated) or haploidentical related donors, which are better than mismatched, unrelated donors. However, the arrival of new highly effective and low toxic immunotherapeutic or targeted therapies inevitably will relegate allo-SCT to fit patients who fail these treatments, which would then be administered far away from the optimal timing.

## Figures and Tables

**Table 1:** Main characteristics of patients at diagnostic and before allo-SCT.

Characteristics at Diagnosis	*N* (%)	Missing Data (%)
Median age, years (range)	52 (31–67)	0 (0%)
Sex (M/F)	108 (80%)/27 (20%)	0 (0%)
Ann Arbor stage:		2 (1%)
I–II	8 (6%)	-
III–IV	125 (94%)	-
B-symptoms:	44 (38%)	20 (15%)
CNS involvement:	2 (2%)	7 (5%)
Bone marrow involvement:	98 (75%)	4 (3%)
Number of extranodal sites:		7 (5%)
0–1	81 (63%)	-
>1	47 (37%)	-
Mantle cell lymphoma histology:		33 (24%)
Indolent [17]	7 (7%)	-
Classic	67 (66%)	-
Blastic	28 (27%)	-
Characteristics at allo-SCT		-
Median previous lines of therapy (range)	2 (1–8)	2 (1%)
Previous ASCT	66 (49%)	0 (0%)
Previous ibrutinib	19 (14%)	2 (1%)
Median months from diagnosis to allo-SCT (range)	33 (3–164)	0 (0%)
Median age, years (range)	56 (32–70)	0 (0%)
Age >60 years	36 (27%)	0 (0%)
Donor type:		0 (0%)
HLA-id sibling	74 (55%)	-
HLA-id unrelated	29 (21%)	-
Haploidentical	18 (13%)	-
Mismatch unrelated	14 (10%)	-
Median donor age (range)	46 (19–72)	2 (1%)
Donor sex:		0 (0%)
Male	82 (61%)	-
Female	53 (39%)	-
ECOG PS:		12 (9%)
0	74 (60%)	-
1	43 (35%)	-
2–4	6 (5%)	-
HCT-CI:		11 (8%)
0–1	70 (56%)	-
2	28 (23%)	-
≥3	26 (21%)	-
Disease status:		1 (1%)
CR	86 (64%)	-
PR	35 (26%)	-
SD/PD	13 (10%)	-
NE	1 (1%)	-
Conditioning regimen:		0 (0%)
Myeloablative	20 (15%)	-
RIC	115 (85%)	-
Myeloablative conditioning:		0 (0%)
Cy + TBI	10 (50%)	-
FLUBU	5 (25%)	-
FLUMEL	1 (5%)	-
BEAM	1 (5%)	-
Other	3 (15%)	-
RIC:		0 (0%)
FLUMEL	90 (78%)	-
FLUBU	15 (13%)	-
Cy + TBI	4 (4%)	-
Other	6 (5%)	-
GVHD prophylaxis:		0 (0%)
CsA/Tacro-MTX	45 (33%)	-
CsA /Tacro-MMF	31 (23%)	-
Timoglobulin based prophylaxis	24 (18%)	-
Tacro-sirolimus	19 (14%)	-
Cy-post	12 (9%)	-
Other	4 (3%)	-
CMV recipient/donor relation:		2 (1%)
R−/D−	12 (9%)	-
R−/D+	11 (8%)	-
R+/D−	35 (26%)	-
R+/D−	75 (56%)	-
Stem cell source:		1 (1%)
PB	127 (95%)	-
BM	5 (4%)	-
UCB *	2 (2%)	-
Median CD34 + cells (range) (×10^6^/kg)	5.1 (0.1–18.1)	5 (4%)

M: male, F: female, CNS: central nervous system, allo-SCT: allogeneic stem cell transplantation, ASCT: autologous stem cell transplantation, HLA-id: HLA identical, ECOG PS: Eastern Cooperative Oncology Group performance status, HCT-CI: hematopoietic cell transplantation-specific comorbidity index, CR: complete response, PR: partial response, SD/PD: stable disease/progression of disease, NE: not evaluable, RIC: reduced-intensity conditioning, GVHD: graft versus host disease, CsA: cyclosporine A, Tacro: tacrolimus, MTX: methotrexate, MMF: Mofetil mycophenolate, Cy: Cyclophosphamide, CMV: cytomegalovirus, R: receptor, D: donor, PB: peripheral blood, BM: bone marrow, UCB: umbilical cord blood. * Included one as haploidentical and the other as mismatch unrelated.

**Table 2:** Analysis of clinical factors influencing non-relapse mortality.

Characteristics	Day100-NRM (%)	*p*
Age at allo-SCT:		1
18–56	14 (20%)	
>56 years	12 (19%)	
Months from diagnosis to allo-SCT:		0.51
0–33	11 (16%)	
>33	15 (22%)	
Previous lines:		0.65
0–2	13 (17%)	
>2	12 (21%)	
Previous ASCT:		0.008
No	7 (10%)	
Yes	19 (29%)	
Donor type:		0.38
HLA-id sibling	12 (16%)	
HLA-id unrelated	5 (17%)	
Mismatch unrelated	5 (36%)	
Haploidentical	4 (22%)	
Donor type:		0.2
HLA-id	17 (16%)	
Mismatch related	4 (22%)	
Mismatch unrelated	5 (36%)	
Donor age:		0.38
18–46	15 (22%)	
>46	10 (15%)	
Donor sex:		0.82
Male	15 (18%)	
Female	11 (21%)	
ECOG PS at allo-SCT		0.31
0–1	21 (18%)	
2–4	2 (33%)	
Pretransplant HCT-CI:		0.58
0–2	18 (18%)	
≥3	6 (23%)	
Conditioning regimen:		0.76
Myeloablative	3 (15%)	
RIC	23 (20%)	
GVHD prophylaxis:		0.25
CsA/Tacro-MTX	20 (22%)	
Other	6 (13%)	
CMV recipient/donor relation:		0.26
R−/D−	1 (8%)	
R−/D+	2 (18%)	
R+/D−	4 (11%)	
R+/D−	19 (25%)	
Stem cell source:		0.54
PB	24 (19%)	
BM	1 (20%)	
UCB	1 (50%)	
CD34+ infused cells (range) (×10^6^/kg):		1
0.1–5.1	13 (20%)	
>5.1	13 (20%)	
aGVHD:		<0.001
No aGVHD or grade 1–2	11 (11%)	
Grade 3–4	15 (43%)	

NRM: non-relapse mortality, allo-SCT: allogeneic stem cell transplantation, ASCT: autologous stem cell transplantation, HLA-id: HLA identical, ECOG PS: Eastern Cooperative Oncology Group performance status, HCT-CI: hematopoietic cell transplantation-specific comorbidity index, RIC: reduced-intensity conditioning, GVHD: graft versus host disease, CsA: cyclosporine A, Tacro: tacrolimus, MTX: methotrexate, CMV: cytomegalovirus, R: receptor, D: donor, PB: peripheral blood, BM: bone marrow, UCB: umbilical cord blood, aGVHD: acute GVHD.

**Table 3:** Univariate and multivariate survival analysis.

Univariate Analysis				
Characteristics	5y-EFS (95% CI)	*p*	5y-OS (95% CI)	*p*
Age at allo-SCT:		0.7		0.66
0–60	50% (40–60)		52% (42–63)	
>60	39% (21–57)		42% (24–60)	
Median months from diagnosis to allo-SCT		0.53		0.77
0–33	51% (39–63)		52% (40–64)	
>33	44% (31–56)		47% (34–60)	
Donor type:		0.032		0.2
HLA-id sibling	52% (40–64)		55% (43–67)	
HLA-id non-related	50% (31–69)		49% (30–68)	
Mismatch non-related	21% (0–45)		35% (7–63)	
Haploidentical	44% (21–67)		42% (17–66)	
Mismatch:		0.018		0.044
Yes	35% (18–52)		39% (20–57)	
No	51% (41–61)		53% (43–63)	
ECOG PS at allo-SCT:		0.035		0.021
0–1	51% (42–60)		54% (45–63)	
2–4	0% (NA)		0% (NA)	
HCT-CI:		0.41		0.42
0–2	49% (39–60)		52% (42–63)	
3 or more	31% (13–50)		35% (16–54)	
Previous lines of therapy:		0.11		0.4
1–2	55% (43–66)		55% (43–66)	
>2	37% (24–50)		42% (28–56)	
Previous ASCT:		0.061		0.02
Yes	39% (27–52)		39% (25–52)	
No	55% (43–67)		59% (48–71)	
Previous ibrutinib:		0.66		0.89
Yes	44% (18–69)		28% (0–69)	
No	48% (39–57)		50% (41–60)	
Conditioning regimen:		0.69		0.99
Myeloablative	48% (26–70)		48% (25–70)	
RIC	47% (38–57)		50% (40–60)	
Response pre-allo-SCT:		0.005		0.004
CR	54% (43–65)		58% (47–69)	
PR	40% (23–57)		43% (25–60)	
SD/PD	15% (0–35)		15% (0–35)	
GVHD prophylaxis:		0.021		0.008
CsA/Tacro-MTX	62% (47–76)		66% (52–80)	
Other	40% (30–51)		41% (30–52)	
Time-period:		0.02		0.023
1995–2005	69% (51–87)		69% (51–87)	
2006–2020	42% (32–52)		45% (35–55)	
Time-period:		0.023		0.03
1995–2005	69% (51–87)		69% (51–87)	
2006–2011	33% (20–47)		38% (24–52)	
2012–2020	49% (37–62)		50% (37–64)	
Time-dependent variables (univariate)	EFS	*p*	OS	*p*
	HR (95% CI)		HR (95% CI)	
Grade 3–4 aGVHD	5.1 (3.2–8.1)	<0.001	6 (3.7–9.8)	<0.001
Chronic GVHD	1 (0.5–2)	0.97	1 (0.5–2)	0.92
Multivariate analysis				
Grade 3–4 aGVHD:	7.6 (4.5–12.8)	<0.001	8.9 (5.1–15.3)	<0.001
Mismatch non-related:	3 (1.5–6.2)	0.002	-	-
Time-period 2006–2020:	2.7 (1.1–6.4)	0.023	3.2 (1.3–8)	0.014

EFS: event-free survival, OS: overall survival, allo-SCT: allogeneic stem cell transplantation, HLA-id: HLA identical, ECOG PS: Eastern Cooperative Oncology Group performance status, HCT-CI: hematopoietic cell transplantation-specific comorbidity index, ASCT: autologous stem cell transplantation, RIC: reduced-intensity conditioning, CR: complete response, PR: partial response, SD/PD: stable disease/progression of disease, GVHD: graft versus host disease, CsA: cyclosporine A, Tacro: tacrolimus, MTX: methotrexate, aGVHD: acute GVHD.

**Table 4:** Analysis of patient characteristics according to time periods.

Characteristics	Global Series (*N* = 135)	2006–2020	1995–2005	*p*
		(*N* = 109)	(*N* = 26)	
Median age at allo-SCT, years (range)	56 (32–70)	57 (32–70)	52 (34–68)	0.047
Median months diagnosis to allo-SCT (range)	33 (3–164)	39 (3–164)	13 (5–84)	0.001
Frontline therapy cytarabine-based	93 (70%)	76 (70%)	17 (68%)	0.81
More than 33 months from diagnosis to allo-SCT	67 (50%)	46 (42%)	5 (19%)	<0.001
Donor type:				<0.001
HLA-id sibling	74 (55%)	50 (46%)	24 (92%)	
HLA-id non-related	29 (21%)	28 (26%)	1 (4%)	
Haploidentical	18 (13%)	18 (16%)	0 (0%)	
Mismatch non-related	14 (10%)	13 (12%)	1 (4%)	
ECOG PS at allo-SCT:				1
0–1	117 (95%)	98 (95%)	19 (95%)	
2–4	6 (5%)	5 (5%)	1 (5%)	
HCT-CI at allo-SCT:				0.11
0–1	70 (56%)	56 (53%)	14 (78%)	
2	28 (23%)	25 (24%)	3 (17%)	
3 or more	26 (21%)	25 (24%)	1 (6%)	
>2 previous lines:	57 (43%)	52 (48%)	5 (20%)	0.013
Previous ASCT:	66 (49%)	61 (56%)	5 (19%)	0.001
Response pre-allo-SCT:				0.92
CR	86 (64%)	70 (64%)	16 (61%)	
PR	35 (26%)	28 (26%)	7 (27%)	
SD/PD	13 (10%)	101 (9%)	3 (11%)	
NE	1 (1%)	1 (1%)		
Conditioning:				0.067
Myeloablative	20 (15%)	13 (12%)	7 (27%)	
RIC	115 (85%)	96 (88%)	19 (73%)	
GVHD prophylaxis:				<0.001
CsA/Tacro-MTX	45 (33%)	81 (74%)	9 (35%)	
Other	90 (67%)	28 (26%)	17 (65%)	
Donor median age (range)	46 (19–72)	45 (19–72)	53 (25–70)	0.052
Stem cell source:				0.26
PB	127 (95%)	104 (96%)	23 (88%)	
BM	5 (4%)	3 (3%)	2 (8%)	
UCB	2 (2%)	1 (1%)	1 (4%)	
Median de CD34 + cells (range) (×10^6^/kg)	5.1 (0.1–18.1)	5.1 (0.1–13)	4.3 (2.1–18.1)	0.45
aGVHD at + 100:	74 (55%)	70 (64%)	13 (50%)	0.5
aGVHD at + 100:				0.37
No aGVHD	60 (45%)	39 (36%)	13 (50%)	
1–2	42 (31%)	38 (35%)	8 (31%)	
3–4	32 (24%)	32 (29%)	5 (19%)	
Chronic GVHD (%):	48 (36%)	36 (33%)	12 (46%)	0.26
Chronic GVHD (%):				0.59
No	87 (64%)	73 (68%)	14 (54%)	
Mild	17 (13%)	14 (13%)	3 (12%)	
Moderate	15 (11%)	11 (10%)	4 (16%)	
Severe	14 (10%)	10 (9%)	4 (16%)	
Overall NRM (%):	61 (45%)	54 (49%)	7 (27%)	0.048
NRM at +100 (%):	27 (20%)	24 (22%)	3 (11%)	0.28
NRM at 1 year (%)	45 (33%)	40 (37%)	5 (19%)	0.11
Causes of death:				0.16
aGVHD	32 (45%)	30 (49%)	2 (20%)	
Infection	15 (21%)	11 (18%)	4 (40%)	
Lymphoma	12 (17%)	9 (15%)	3 (30%)	
Other	12 (17%)	11 (18%)	1 (10%)	
Response post-allo-SCT:				0.12
CR	110 (81%)	88 (81%)	22 (85%)	
PR	6 (4%)	6 (5%)	0 (0%)	
SD/PD	6 (4%)	4 (4%)	2 (8%)	
NE	13 (10%)	11 (10%)	2 (8%)	

Allo-SCT: allogeneic stem cell transplantation, HLA-id: HLA identical, ECOG PS: Eastern Cooperative Oncology Group performance status, HCT-CI: hematopoietic cell transplantation-specific comorbidity index, ASCT: autologous stem cell transplantation, CR: complete response, PR: partial response, SD/PD: stable disease/progression of disease, NE: not evaluable, RIC: reduced-intensity conditioning, GVHD: graft versus host disease, CsA: cyclosporine A, Tacro: tacrolimus, MTX: methotrexate, PB: peripheral blood, BM: bone marrow, UCB: umbilical cord blood, aGVHD: acute GVHD; NRM: non-relapse mortality.

## Data Availability

The data presented in this study are available on request from the corresponding author.
